# QuickStats

**Published:** 2014-02-14

**Authors:** FROM THE NATIONAL CENTER FOR HEALTH STATISTICS

**Figure:**
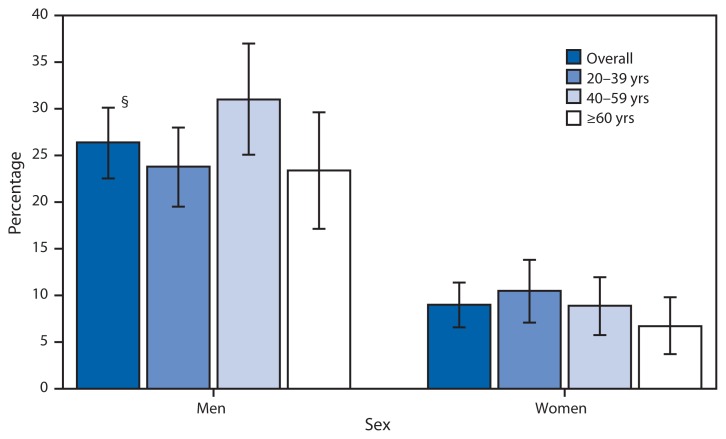
Percentage of Adults Age ≥20 Years with Low Levels of High-Density Lipoprotein (HDL) Cholesterol,* by Age Group and Sex^†^ — National Health and Nutrition Examination Survey, 2011–2012 * Low HDL cholesterol defined as serum HDL cholesterol <40 mg/dL. ^†^ Overall estimates for men and women are age-adjusted by the direct method to the year 2000 Census population using the following age groups: 20–39, 40–59, and ≥60 years. ^§^ 95% confidence interval.

During 2011–2012, an estimated 26.4% of U.S. adult males and 9.0% of females aged ≥20 years had low levels of HDL cholesterol (also known as “good cholesterol”). In all age groups, a higher percentage of men had low levels of HDL cholesterol than women. A higher percentage of men aged 40–59 years had low levels of HDL cholesterol than men aged ≥60 years.

**Source:** Carroll MD, Kit BK, Lacher DA, Yoon SS. Total and high-density lipoprotein cholesterol in adults: National Health and Nutrition Examination Survey, 2011–2012. NCHS data brief no. 132. Hyattsville, MD: US Department of Health and Human Services, CDC; 2013. Available at http://www.cdc.gov/nchs/data/databriefs/db132.htm.

**Reported by:** Margaret D. Carroll, MSPH, mdc3@cdc.gov, 301-458-4136; Steven M. Frenk, PhD.

